# Factors associated with the time to cessation of breastfeeding among mothers who have index children aged two to three years in Debre Markos, northwest Ethiopia: a retrospective follow up study

**DOI:** 10.1186/s12887-018-1012-3

**Published:** 2018-02-22

**Authors:** Melkamu Tamir Hunegnaw, Kassahun Alemu Gelaye, Bekri Mohammed Ali

**Affiliations:** 1 Department of Human Nutrition, College of Medicine and Health Sciences, the University of Gondar, P.O. Box 196, Gondar, Ethiopia; 2 Department of Epidemiology and Biostatistics, Institute of Public Health, College of Medicine and Health Sciences, The University of Gondar, Gondar, Ethiopia

**Keywords:** Ethiopia, Cessation of breastfeeding, Factors

## Abstract

**Background:**

Breastfeeding of children, which needs to continue until two years and beyond, is one of the essential requirements for child survival. However, in Ethiopia there is scarcity of literatures on the duration of breastfeeding. Therefore, the aim of this study was to assess the rate of cessation of breastfeeding among mothers with index children aged 2 to 3 years, northwest Ethiopia.

**Methods:**

A retrospective follow-up study was conducted at Debre Markos town from March 1, 2014 to March 30, 2016. A total of 500 mother-child pairs were selected using the systematic random sampling method by moving from house to house with an interval of three eligible houses. A structured questionnaire was used to collect data. The Cox regression model was employed to identify the predictors of breastfeeding cessation.

**Results:**

The proportion of women breastfeeding until 2 years was 13.70 per 1000 person- months. HIV-positive mothers decreased the time of breastfeeding by 3.4 times compared to HIV-negative mothers (AHR = 3.41, 95% CI: 1.96, 5.94). Government employee mothers decreased the time of breastfeeding by 2.8 times compared to housewives (AHR = 2.8, 95% CI: 1.80, 4.40).Better education increased the time of breastfeeding (AHR = 0.45, 95% CI: 0.24, 0.58). Number of children, family income, and place of delivery were the other significant predictors of time to cessation of breastfeeding (*p* < 0.05).

**Conclusion:**

In this study, the rate of cessation of breastfeeding was good. HIV negative mothers, government employment, number of children, place of delivery, and family monthly income were significant predictors to the time of breastfeeding cessation. Therefore, family planning and breastfeeding education in health institutions are essential to increase breastfeeding duration.

## Background

Breast milk, the first natural food for children, has nutritional, immunological, developmental, and psychological advantages [1]. The World Health Organization (WHO) recommends that children should be breastfed until 2 years of age or beyond [2].The promotion of children breastfeeding until 2 years is one of the indicators of appropriate child feeding practices [3].

Although breastfeeding practice is universal in Africa, where more than 90% of the mothers breastfeed, some mothers initiate the practice late; others do not do it exclusively, and still others cut the duration to less than two years [4]. In the continent, late cessation of breastfeeding (CBF) has greater effects on child morbidity and mortality [5].The problem of malnutrition begins early in life during the first two years due to suboptimal breastfeeding [6]. A meta- analysis study showed that late CBF was associated with elevated risk of pneumonia [7].

The time to breastfeeding cessation varies from country to country. For instance, in America, Iran, and Italy, 60%, 57%, and 12% [8–10] of the mothers ceased breastfeeding before the children were two years of age, respectively. In China, the median breastfeeding duration was 6.0 months in urban groups and 8.0 months in rural groups [11], and it was 8.6 months in the United Arab Emirates [12]. In Pakistan more than half of the mothers (54%) ceased breastfeeding before the children were 6 months of age [13]. In Tanzania, 94.0% of the infants were breastfed till 12–15 months, but the proportion of breastfeeding decreased to 51.1% at 20–23 months of age [14].

Studies done in Lithuania and Norway showed that the time to CBF was associated with maternal factors, such as age, ethnicity, religion, and marital status [15, 16]. Studies done in Brazil, Nigeria, and Kuwait showed that the time to CBF was associated with child sex, place of delivery, maternal education, maternal employment, and family monthly income [17–19]. In addition, health and health service related factors, maternal and child illness [15], multiple births, breastfeeding experience [5], HIV status of the mother [5, 20], mode of delivery [21], place and attendant of delivery, birth interval, and antenatal care [22] were also factors influencing the time to CBF. Similarly, the time to CBF was related to support from father [23], postpartum employment [17], mother’s attitude and knowledge about breastfeeding [18].

The Federal Ministry of Health of Ethiopia has developed a guideline on infant and young child feeding practices [6]. However, the duration of breastfeeding has decreased from time to time [24]. Therefore, this study aimed to assess the rate and predictors of the time to CBF, using survival analysis among mothers who had index children aged two to three years at Debre Markos, northwest Ethiopia.

## Methods

### Study design and period

A quantitative community-based retrospective follow-up study was conducted between March 2014 and March 2016 to assess the rate of CBF before two years and associated factors among mothers who had index children aged two to three years.

### Study setting

Debre Markos town is located at 300 km northwest of Addis Ababa, the capital of Ethiopia. In Debre Markos, there are seven kebeles (the lowest local administrative units). The total population of the town is 101,582, (52,833 female and 48,749 are male). In the town, there are 23,956 women in the reproductive age group (15–49 years) and 2310 children two to three years of age [24]. In the town one referral hospital, three health centers, and two nongovernmental organization clinics provide health care services to the residents. 

### Sample size, sampling technique, and procedures

For determining the sample size, a single population formula was used with an assumption of 95% confidence interval, marginal error of 5%,and 18% as the proportion of CBF before two years of age [24]. Adding 10% to account for a non-response rate and a design effect of 2 gave us the final sample of 500mother-childpairs.By taking the ratio of the total mother-child pairs of 1632 in five selected kebeles a total sample size of 500 was attained with a sample interval of three.

In the five selected kebeles, a total of 1632 (273,597,300,229 and 233 in each kebele) eligible mother-child pairs were found, out of which 500(84, 183, 92, 70 and 71) eligible participants were selected for each kebele, using the population proportion formula. Mother-child pairs were selected using the systematic random sampling technique with a sample interval of three eligible households. To get eligible mother-child pairs, we moved from block to block of each selected kebele and every household was visited until the required sample size was secured.

### Data collection

Data were collected using an interviewer-administered structured questionnaire. The questions were drawn from the literature in the Ethiopian Demographic Health Survey, and we also used some literature on breastfeeding written in the Ethiopian context. The questionnaire comprised socio-demographic, health service, and obstetric related components.

The survival data were collected from mothers with index children aged two to three years. The mothers were asked the date on which they ceased breastfeeding, which was the event of interest. The date of birth of the index child was taken as the starting point of the retrospective follow-up study. The length of time was measured in months (from birth to 3 years of age) and was taken to be the survival time for those who had experienced the event of interest. A mother who ceased breastfeeding before two years was an event of interest, and those who were breastfed during data collection were considered as right censored.

Possible predictors of time to CBF, such as socio-demographic variables, like number of children, birth interval, mode of delivery, breastfeeding experience and place of delivery were studied. In addition to these, ANC follow up, attendant of delivery, breastfeeding counseling before and after delivery, HIV status of the mother, knowledge and attitude about breastfeeding were the other potential factors evaluated in terms of their association with the time to CBF.

### Data quality control

In order to maintain the quality of data, the principal investigator trained the five data collectors and one supervisor for one day. A pretest was conducted on 20 mothers from a non-selected kebele. On-site supervision was performed and each copy of the questionnaire was checked for completeness and accuracy before data entry, and incomplete questions were excluded.

### Definitions

#### Early cessation of breastfeeding

Mothers stoppages of breastfeeding before their children are two years of age.

#### Knowledge

Mothers’ awareness about the advantages and duration of breastfeeding.

#### Adequate knowledge

If a mother answered at least nine of the twelve knowledge assessment questions correctly.

#### Favorable attitude

If a mother responded positively to at least nine of the twelve attitude assessment questions.

### Statistical analysis

Data were entered, coded and cleaned using Epi-info version7.0statistical software and were then exported to SPSS version 20 for further analysis. The Kaplan-Meier curve was used to measure the probability of surviving the breastfeeding duration.

Incidence rate was calculated as the number of events over the person-months of follow- up. Both bivariate and multivariate Cox proportional regression models were used to identify factors that affected the CBF before two years. Variables with *P*-value < 0.2 in the bivariate Cox regression model were entered spontaneously into the multivariate Cox regression model to measure the effect of each variable on the hazard function after adjusting the effects of other variables using the Backward LR method. Variables with *P*-value< 0.05 in the multivariate Cox regression analysis were considered as statistically significant for the CBF before two years.

## Results

### Socio-demographic, health, and health service-related characteristics

In the study, a total of 500 mother-child pairs were included. Of these, 483 (96.6%) were followed retrospectively. The mean age of the mothers was 28.59 (± 4.95) years, while that of the children was 30.11 (±4.15) months. In this study, the majority of the mothers were married; they were Amhara by ethnicity. Almost half of the mothers were housewives, and one-third completed secondary school [Table 1].

**Table 1:** Socio-demographic characteristics of mothers who have index children aged two to three years, northwest Ethiopia 2016 (*n* = 483)

Variables	Number	Percent
Age of mother		
15–19	4	0.8
20–24	86	17.8
25–29	199	41.2
30–34	130	26.9
≥ 35	64	13.3
Marital status		
Married	426	88.2
Divorced	13	2.7
Widowed	44	9.1
Sex of index child		
Male	263	54.5
Female	220	45.5
Family size		
≤ 4	266	55.1
> 4	217	44.9
Number of children		
≤ 3	419	86.7
> 3	64	13.3
Maternal education		
non educated or Informal education	91	18.8
Primary education	93	19.3
Secondary education	141	29.2
Certificate, and above	158	32.7
Educational status of father		
Not educated or Informal education	88	18.2
Primary education	70	14.5
Secondary education	119	24.6
Certificate and above	206	42.7
Maternal employment		
House wife	252	52.2
Government	107	22.2
Private	113	23.4
Private organization	11	2.3
Family monthly income		
≤ 29.5$	76	15.7
29.6–63.6$	89	18.4
63.7–106.8$	105	21.7
106.9–161.4$	81	16.8
≥ 161.41$	132	27.3

More than half of the mothers (62%) had adequate knowledge, whereas half of them had favorable attitude towards breastfeeding in general, and the majority preferred breastfeeding to formula feeding [Table 2].

**Table 2:** Health and health service related characteristics of mothers who have index children aged two to three years at Debre Markos, northwest Ethiopia, 2016 (n = 483)

Characteristics	Number	Percent
Antenatal care		
Yes	455	94.2
No	28	5.8
BF counseling during ANC		
Yes	336	69.6
No	147	30.4
Place of delivery		
At home	53	11.0
Government hospital	326	67.5
Government health center	95	19.7
Non government health facility	9	1.8
Mode of delivery		
Vaginal	441	91.3
Cesarean section	42	8.7
Attendant of the delivery		
Health profession	435	90.1
Relative/Friends	48	9.9
Breastfeeding counseling after delivery		
Yes	370	76.6
No	113	23.4
Breastfeeding experience		
Yes	274	57.7
No	209	43.3
Maternal HIV status		
Positive	28	5.8
Negative	455	94.2
Knowledge about breastfeeding		
Adequate	297	61.5
Inadequate	186	38.5
Attitude about breastfeeding		
Favorable	252	52.2
Unfavorable	231	47.8

### Survival analysis for breastfeeding cessation

**Fig. 1:**
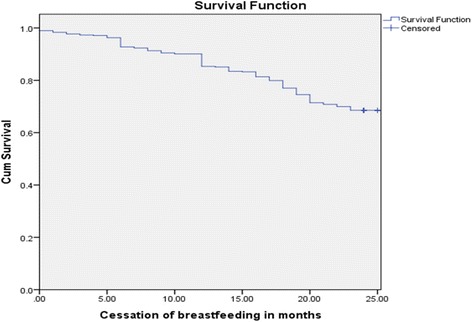
Survival of breastfeeding among mothers who have index children aged two to three years at Debre Markos, northwest Ethiopia, 2016

The overall person-time of CBF was 11,181 person months. The overall incidence rate of CBF before 2 years of age was 13.70 per 1000 person-months (95% CI: 27.3–35.8). The cumulative survival probability in life table indicated that the percentage of children who remained on breastfeeding for the first two years was 68.5% [Fig. 1].

### Multivariate survival analysis

In the bivariate Cox regression model, age, marital status, educational status, occupation of mother, place of delivery, HIV status of the mother, family monthly income, and number of children were independent predictors of CBF before two years of age(*p* < 0.2). In the multivariate Cox regression model, maternal education, maternal occupation, place of delivery, HIV status of the mother, family monthly income, and number of children were significantly associated with time to cessation of breastfeeding at 95% confidence level (*p* < 0.05). 

In this study, mothers who were government employees decreased the time of breastfeeding by 2.8 times compared to housewife mothers (AHR = 2.81, 95% CI: 1.80–4.38). HIV positive mothers deceased the time of breastfeeding almost by 3.4 times compared to HIV-negative mothers (AHR = 3.42, 95% CI: 1.96–5.94). Mothers with less than three children increased the time of breastfeeding by 57% compared to mothers who had more than three children (AHR = 0.43, 95% CI: 0.28–0.65). Mothers who had certificate and above educational qualification increased the time of breastfeeding almost by 55% compared to less educated mothers (AHR = 0.45, 95% CI: 0.24, 0.85) [Table 3].

**Table 3:** Bivariate and multivariate cox regression of CBF among mothers who have index children aged two to three years Debre Markos, northwest Ethiopia 2016 (n = 483)

Variables	Cessation of breastfeeding			
	Yes (%)	No (%)	CHR (95% CI)	AHR (95% CI)
Age of mother (in years)				
< =24	18 (20.0)	72 (80.0)	1	1
25–29	61 (30.6)	138 (69.4)	1.59 (0.94,2.69)	1.27 (0.73,2.20)
30–34 ≥ 35	48 (36.9)	82 (63.1)	2.05 (1.19,3.53)	1.33 (0.72,2.43)
	26 (40.6)	38 (59.4)	2.48(1.36,4.53)	1.34 (0.67,2.65)
Marital status				
Married	127 (29.8)	299 (70.2)	0.61 (0.38,1.00)	0.77 (0.43,1.37)
Divorced	7 (53.8)	6 (46.20)	1.20 (0.51, 2.87)	0.44 (0.32,2.22)
Widowed	19 (43.2)	25 (56.8)	1	1
Maternal education				
Non educated and Informal Education	32 (35.2)	59 (64.8)	1	*1*
Primary education	24 (25.8)	69 (74.2)	0.66 (0.39,1.13)	0.72 (0.42,1.26)
Secondary education	38 (26.95)	103 (73.1)	0.69 (0.44,1.12)	0.59 (0.34,1.03)
Certificate and above	59 (37.3)	99 (62.7)	1.00 (0.65,1.54)	0.45 (0.24, 0.85)^*^
Educational status of father				
Non educated or Informal Education	30 (34.1)	58 (65.9)	1	1
Primary education	16 (22.9)	54 (77.1)	0.65 (0.36,1.17)	1.04 (0.52, 2.07)
Secondary education	30 (25.2)	89 (74.8)	0.65(0.39,1.08)	0.93 (0.49,1.77)
Certificate and above	153 (54.3)	129 (45.7)	1.06 (0.69,1.61)	1.04 (0.53, 2.05)
Maternal employment				
House wife	64 (25.4)	188 (74.6)	1	*1*
Government	58 (54.2)	49 (45.8)	2.46 (1.72,3.51)	2.81(1.80, 4.38)^*^
Private organization	2 (18.2)	9 (81.2)	0.62 (0.15,2.53)	0.76 0.18, 3.13)
Private(self) work	29 (25.7)	84 (74.3)	1.01 (0.65,1.57)	0.95 (0.61, 1.49)
Monthly family income				
≤ 29.5$	33(43.4)	43 (56.6)	1	*1*
29.6–63.6$	17 (19.1)	72 (80.9)	0.36 (0.20, 0.64)	0.29(0.16, 0.54)^*^
63.7–106.8$	27 (25.7)	78 (74.3)	0.50 (0.30, 0.83)	0.40 (0.24, 0.68)^*^
106.9–161.4$	17 (21.0)	64 (79.0)	0.38 (0.22, 0.69)	0.29 (0.16, 0.55)^*^
≥ 161.41$	59 (44.7)	73 (55.3)	0.97 (0.63,1.48)	0.48 (0.29, 0.81)^*^
Antenatal care (ANC)				
Yes	144 (31.6)	311 (68.4)	**1**	**1**
No	9 (32.1)	19 (67.9)	1.08 (0.55, 2.11)	1.13 (0.53,2.38)
Family size				
≤ 4	78 (29.3)	188 (70.7)	0.8 (0.58,1.10)	0.88 (0.58,1.34)
> 4	75 (34.6)	142 (65.4)	1	**1**
Place of delivery				
At home	10 (18.9)	43 (81.1)	1	*1*
Government hospital	110 (33.7)	216 (66.3)	1.93 (1.01,3.68)	2.30 (1.15, 4.62)*
Health center, post	33 (31.7)	71 (68.3)	1.75 (0.86,3.55)	2.04 (0.96, 4.32)
Attendant of delivery				
Health professional	144 (33.1)	291 (66.9)	1	1
Relative/friend	9 (18.75)	39 (81.25)	0.53 (0.27, 1.04)	1.08 (0.26, 4.38)
Mode of delivery				
Cesarean section	18 (42.8)	24 (57.2)	1.523 (0.931, 2.49)	1.3 (0.75, 2.24)
Vaginal	135 (30.6)	306 (69.4)	1	1
BF counseling on ANC				
Yes	128 (34.6)	242 (65.4)	1.30 (0.90, 1.87)	1.29 (0.89, 1.87)
No	25 (22.3)	87 (77.7)	1	1
Number of Children				
≤ 3	124 (29.6)	295(70.4)	0.57(0.38, 0.86)	0.43 (0.28, 0.65)^*^
> 3	29 (45.3)	35(54.7)	1	*1*
Maternal HIV status				
Positive	17 (60.7)	11 (39.3)	2.66 (1.61, 4.42)	3.42 (1.96, 5.94)^*^
Negative	136 (29.9)	319 (70.1)	1	*1*
BF Experience				
Yes	83 (30.3)	191 (69.7)	1	1
No	70 (33.5)	139 (66.5)	1.12 (0.82, 1.54)	1.31 (0.93, 1.85)
Attitude of mother				
Favorable	74 (29.4)	178 (70.6)	1	1
Unfavorable	79 (34.2)	152 (65.8)	0.81 (0.59, 1.11)	0.81 (0.88, 1.71)

*Statistically significant at *P* value < 0.05, *1* *BF* Breastfeeding

*CHR* Crude hazard ratio, *AHR* Adjusted hazard ratio

"1" indicates statistically significant variables

## Discussion

In this study, 483 mother-child pairs were followed retrospectively for a total of 11,181 person-months. The incidence rate of breastfeeding cessation before two years of age was 13.70 person-months. One-third of the mothers (32%) ceased breastfeeding before two years of age. This prevalence is higher than the national average [24], but lower than that of a study done in Iran (57%) [9].The variation may be due to the fact that the present study considered only urban residents, while the national study considered both urban and rural residents. But the difference between this and the Iranian study may be due to the socio-demographic variations between the two countries.

This study showed that HIV-positive mothers reduced the time of breastfeeding by 3.4 times compared to HIV-negative mothers. This result corresponds to that of a study done in South Africa [5]. The low breastfeeding duration of HIV-positive mothers might be due to the fear of HIV transmission to their children and mothers’ illness due to HIV/AIDS.

In this study, mothers who were better educated increased the time of breastfeeding compared to less educated mothers. This is in line with other findings in South Africa and Kuwait [5, 19] which showed that educational level of mothers influenced breastfeeding duration. The possible explanation might be when mothers are educated, the knowledge of breastfeeding duration and willingness to continue breastfeeding increases compared to non-educated mothers. But a study conducted in India showed that better-educated mothers reduced the time of breastfeeding than less educated ones [25]; this difference might be due to early introduction of supplementary feeding among more educated mothers leading to reduce the time of breastfeeding.

Mothers who had family monthly income of $29.6–63.6 increased the time of breastfeeding by 51% compared to mothers with lower family monthly income. However, a study done in Pakistan [22] showed that mothers who had lower family monthly income increased the time of breastfeeding more than owners of higher family monthly income. The possible reason for the direct relationship between income and breastfeeding duration might be that mothers who had higher family monthly income could have good knowledge about the advantages of breastfeeding.

In this study, mothers with less than four children increased the time of breastfeeding by 57% compared to mothers who had more than four children. This finding is similar with that of a study done in Bangladesh [21].The possible reason for this is that mothers a lower number of children have enough time to continue breastfeeding.

In our study, government employee mothers were more likely to reduce the time of breastfeeding compared to housewife mothers. This finding is in line with those of studies done in Greece and Australia [16, 26]. The explanation for this finding might be that in Ethiopia government employee mothers return to work within a short time (three months) after delivery. In addition to these reasons, in Ethiopia there are no breastfeeding rooms in working areas. This might be the cause of early termination of breastfeeding for government employee mothers.

In Ethiopia, 15% of the births are delivered at health facilities [24]. This study showed that about 89% of the births at Debre Markos town were delivered at health facilities. Mothers who delivered at health institutions decreased the time of breastfeeding compared to mothers who delivered at home, but a study done in India showed that those mothers who delivered at health facility increased the time of breastfeeding [25].The reason might be in India mothers can get better breastfeeding counseling in the health facilities.

### Strengths and limitations

The strength of this study was that it assessed breastfeeding duration up to two years (most studies assessed for 1 year); this adds significant variables to the CBF and helps determine the rate of breastfeeding proportion (person months).

This study has potential limitations, like recall bias which may possibly result in under or over estimation of the actual breastfeeding durations. This bias was not fully controlled although interviewers were trained to minimize it by encouraging mothers to remember when they had their index children by relating their deliveries with the calendar for local events. Another limitation of this study was that the authors did not account for mothers who had breast pumps. In fact, as breast pumps are not common in Ethiopia.

## Conclusion

The incidence rate of time to CBF before children were two years of age was 13.70 per 1000 person- months (95%CI 27.3–35.8). The mean duration of breastfeeding was longer than in most countries. Family monthly income, educational status of the mother, HIV status of the mother, place of delivery, number of children, and employment of mothers were significantly associated with the time to CBF before two years of age. Therefore, interventions such as family planning and educating HIV positive mothers about the options of breastfeeding are essential to increase breastfeeding duration.

## Abbreviations

AHR: Adjusted Hazard Ratio
ANC: Antenatal Care
BF: Breast Feeding
CBF: Cessation of Breast Feeding
CHR: Crude Hazard Ratio
EDHS: Ethiopian Demographic and Health Survey
IYCF: Infant and Young Child Feeding
NGO: None Governmental Organizations
WHO: World Health Organization
